# A Routine Treatment for Septic Sores and Nile Boils

**Published:** 1919

**Authors:** 


***A Routine Treatment for Septic Sores and Nile Boils.*** By H. WARREN CROWE. *The Lancet*, November 16, 1918.

During the period covered by the author's observations, diphtheria bacilli were rare. He found diphtheria-like organisms in about 5 o/o of all sores examined. These were distinguished from the usual ulcers by their depth, and by a tough, blackened, dry membrane which he succeeded in dislodging only with the help of a digestive ferment. The membrane contained diphtheria-like organisms in large numbers. Although staphylococci could be cultured from almost any open sore, yet vaccines of this organism produced no apparent effect.

It seems probable that the condition dealt with was directly due to some cause associated with a hot, dry climate or with a special dietary, and that, in every case, the lesion thus produced was infected with the prevailing microbes. There does not seem to be sufficient evidence to point to any particular climatic agent or form of diet as the actual cause of septic sores.

The distinctive features of the Nile boil are the initial slough, with its red areola, the extreme tenderness, and the complete absence of pus until the last stage.

After culturing some 30 of these boils, the author states that the Staph. aurens is not the cause of Nile boils, though it may possibly be responsible for the pus formation when that occurs. In every case but one he obtained an organism which at first he took to be a short streptococcus, but which he is now inclined to regard as white staphylococcus allied to the S. epidermidis albus of the skin.

Microscopically, the organism is an irregular coccus almost always seen in closely apposed pairs lying in clumps, and larger than a staphylococcus of the pyogenic type. It does not form chains. On subculture the growth soon becomes turbid.

Its most peculiar property is its extreme toxicity when used as a vaccine.

The optimum dose for each vaccine averages 500,000 germs, and these small doses should be given about every 3 days. The best treatment seems to be to give a combined vaccine containing many strains, both from septic sores and also from Nile boils, in doses of 500,000 streptococci and the same number of the boil staphylococcus.

For use in treatment, the following dilutions are needed; 2 million, 8 million, and 32 million to the 20 minims or cc., according to the syringe likely to be used. These figures denote the numbers of germs of each vaccine.

Vaccine is given in bi-weekly doses of 500,000 — i.e., 0.25 cc. or 5 minims out of the bottle containing the weakest dilution until all lesions are cured, until no fresh ones appear, and until, in the case of sores, no old superficial scars start weeping afresh after an inoculation.

All boils must be incised in such a way as completely to divide the central slough at the earliest possible moment.

Septic sores have a tendency to become covered with a seeping scab. Care should be taken to prevent this by soaking and removing dried serum.

